# Supplementary material for: Notch2 Regulates the Function of Bovine Follicular Granulosa Cells via the Wnt2/β-Catenin Signaling Pathway
Source: Animals (Basel). 2024 Mar 25;14(7):1001. doi: 10.3390/ani14071001 (PMC11010942; doi:10.3390/ani14071001)
Supplement: Supplementary file 1 [file animals-14-01001-s001.zip › animals-2892246-supplementary.pdf]

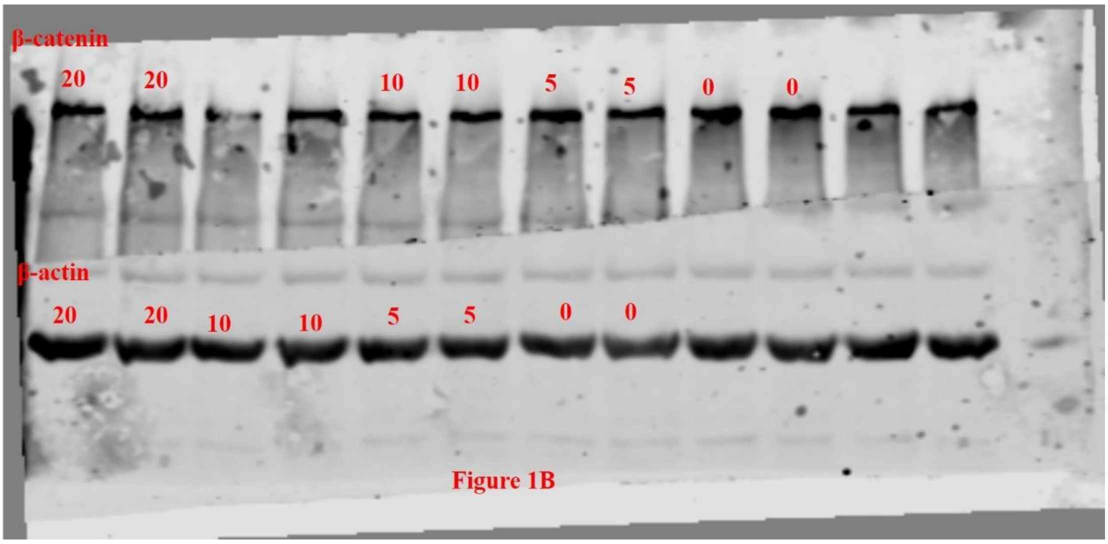


Figure S1: Western blotting gray value statistics of β-catenin protein expression in GCs treated with IWR-1.

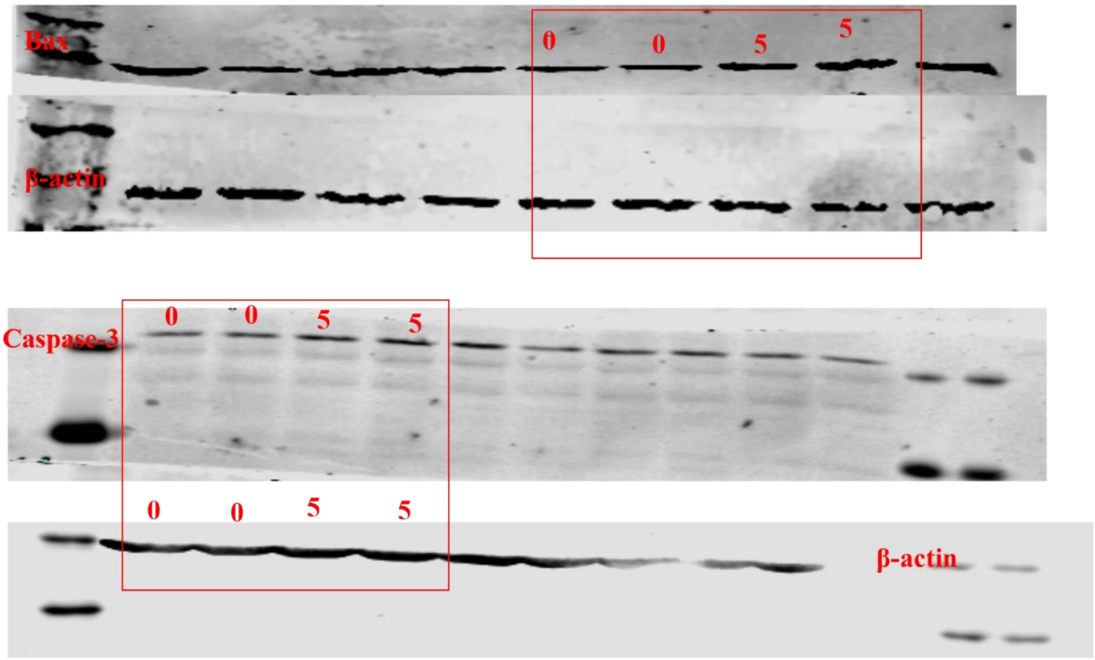


Figure 1F

Figure S2: Western blotting gray value statistics of Bax and Caspase-3 protein expression in GCs treated with IWR-1.

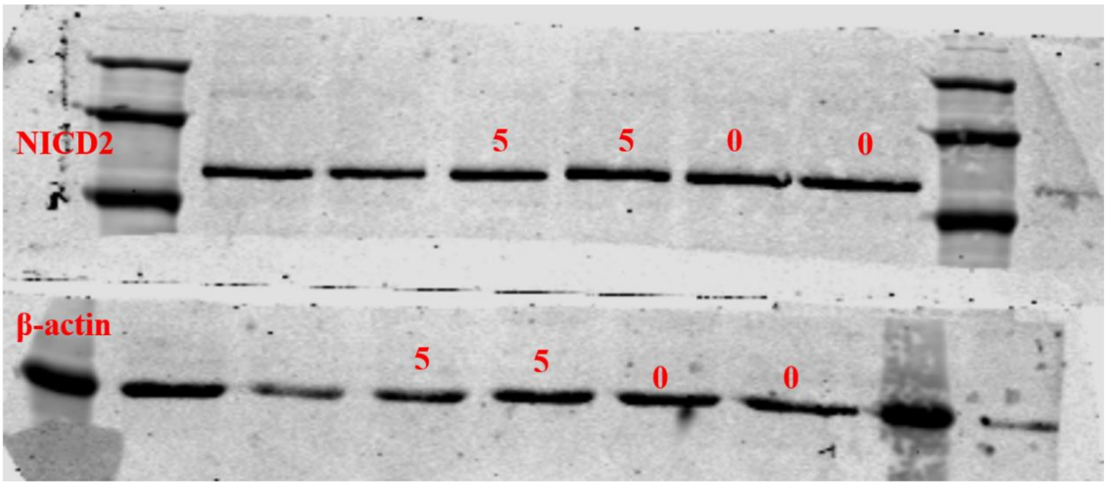


Figure 2A

Figure S3: Western blotting gray value statistics of NICD2 protein expression in GCs treated with IWR-1.

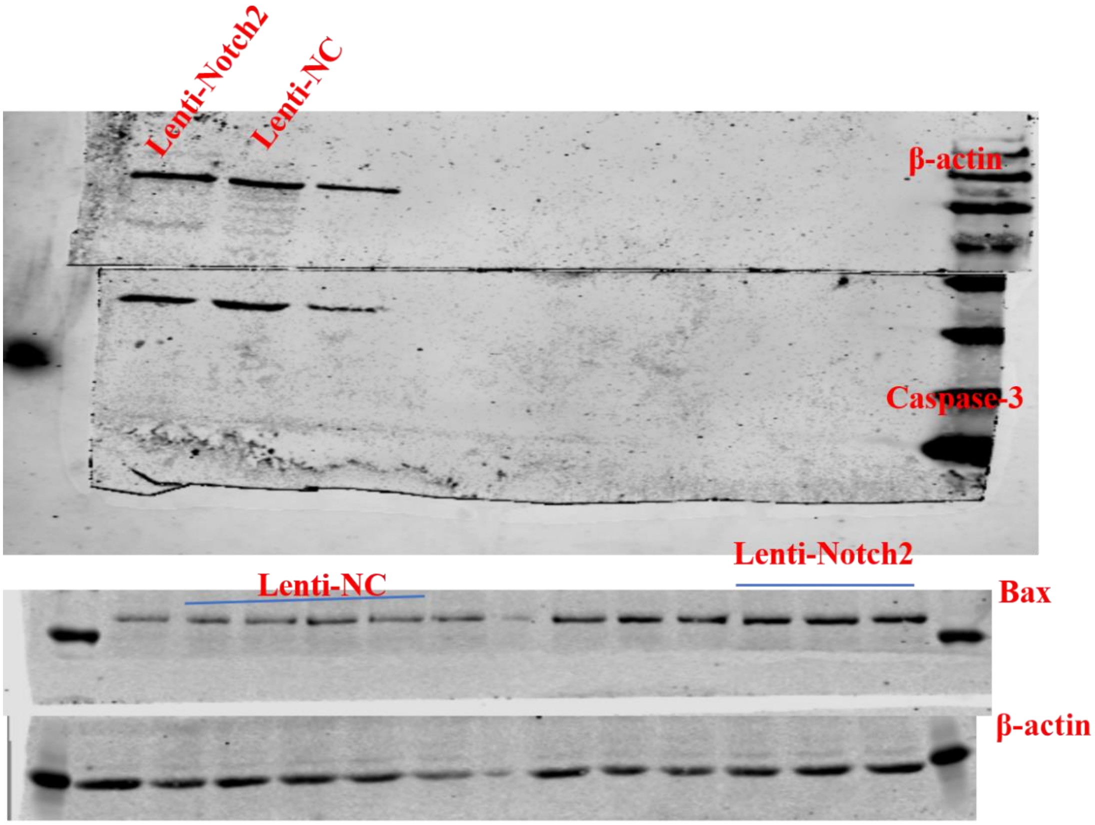


Figure 5A

Figure S4: Western blotting gray value statistics of Bax and caspase-3 protein expression in GCs by *Notch2* silencing.

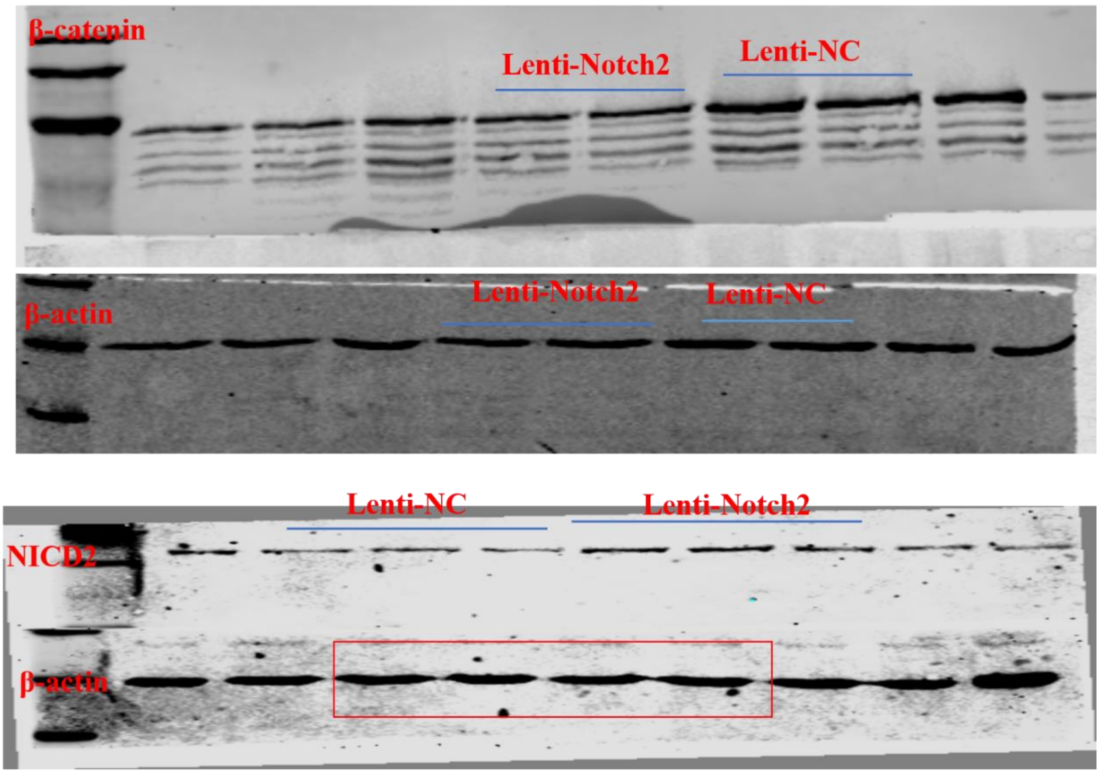


**Figure 6A**

Figure S5: Western blotting gray value statistics of β-catenin and NICD2 protein expression in GCs by *Notch2* silencing.

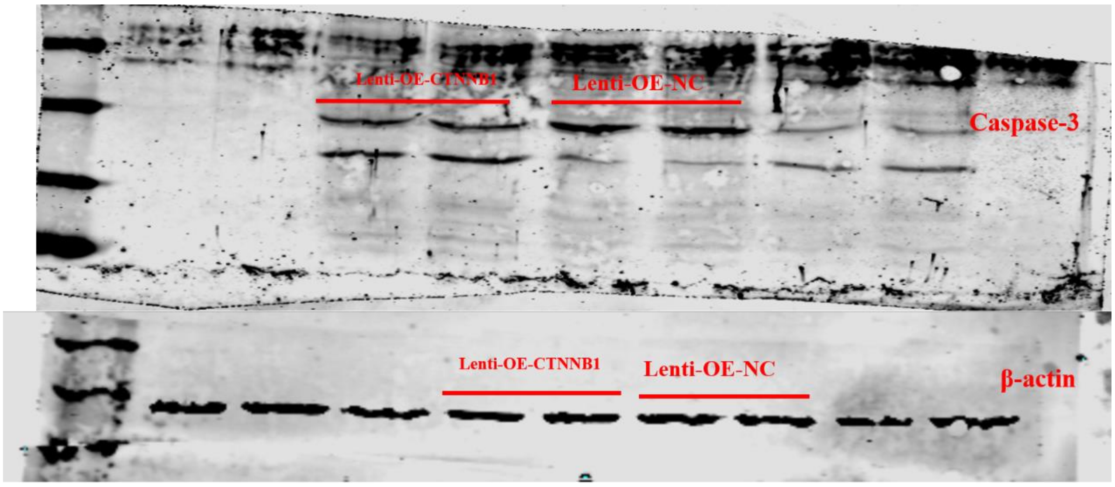


**Figure 9A**

Figure S6: Western blotting gray value statistics of caspase-3 protein expression in GCs by overexpression *CTNNB1*.

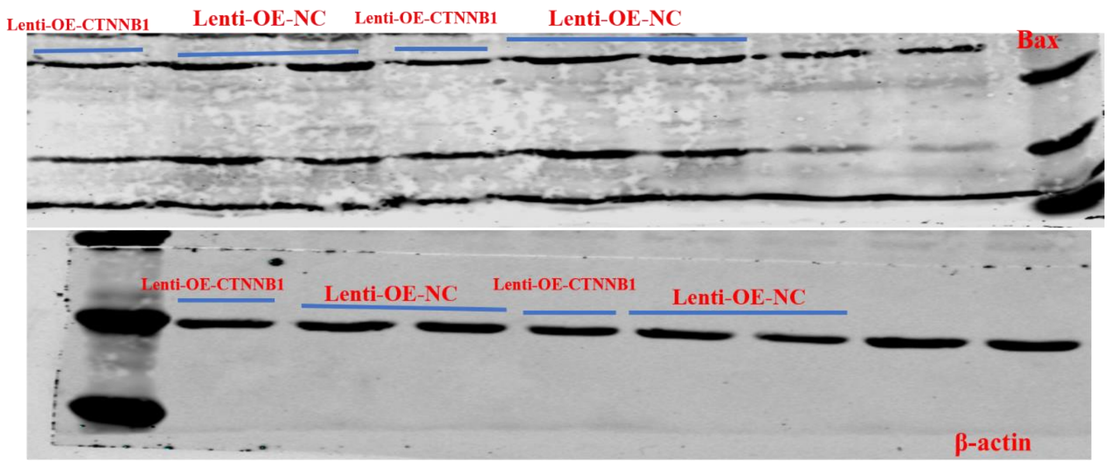


**Figure 9B**

Figure S7: Western blotting gray value statistics of Bax protein expression in GCs by overexpression *CTNNB1*.

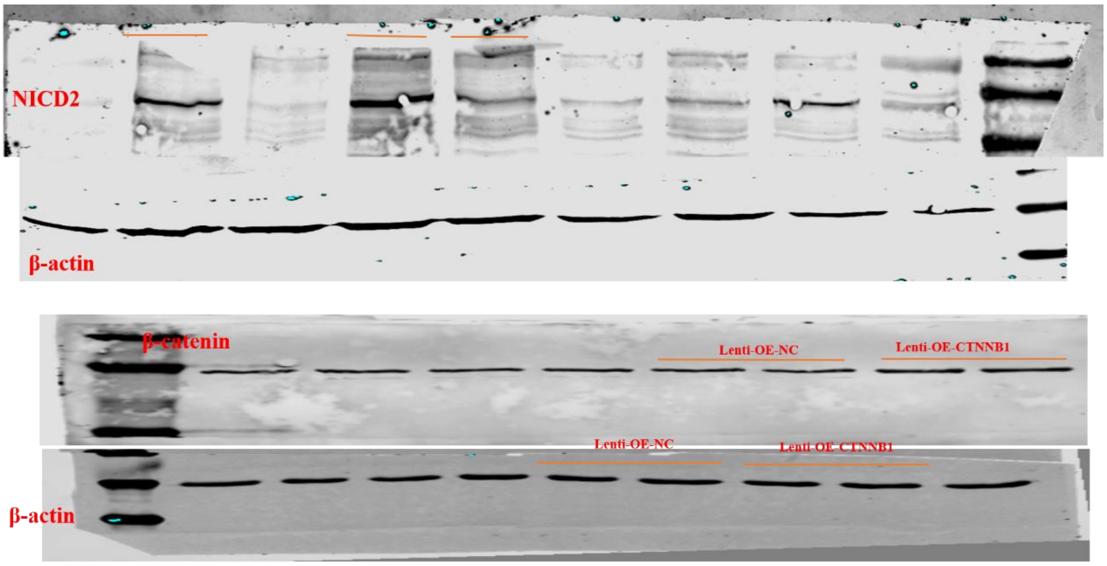


**Figure 11A,B**

Figure S8: Western blotting gray value statistics of β-catenin and NICD2 protein expression in GCs by overexpression *CTNNB1*.

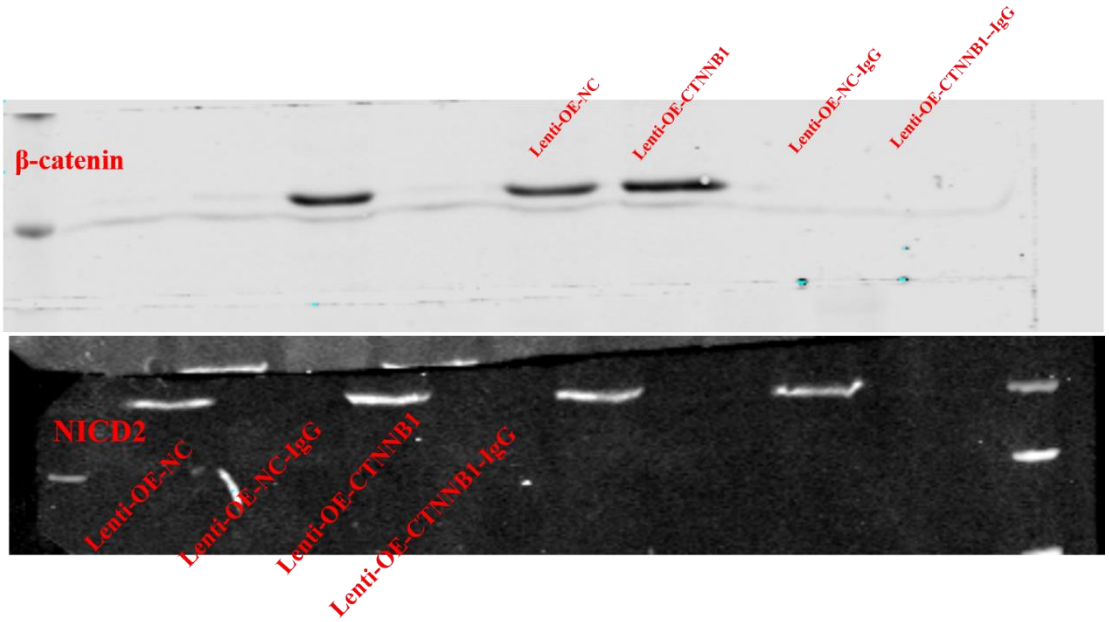


**Figure 11C**

Figure S9: Protein expression of β-catenin and NICD2 in protein complexes in GCs by CO-IP.
